# Cardiovascular Calcifications Are Correlated with Inflammation in Hemodialysis Patients

**DOI:** 10.3390/medicina59101801

**Published:** 2023-10-10

**Authors:** Dorin Dragoș, Delia Timofte, Mihai-Teodor Georgescu, Maria-Mirabela Manea, Ileana Adela Vacaroiu, Dorin Ionescu, Andra-Elena Balcangiu-Stroescu

**Affiliations:** 1 Faculty of Medicine, Carol Davila University of Medicine and Pharmacy, Dionisie Lupu nr 37, Sect 2, 020021 Bucharest, Romaniaileana.vacaroiu@umfcd.ro (I.A.V.);; 2 1st Internal Medicine Clinic, University Emergency Hospital Bucharest, Splaiul Independentei nr 169, Sect 5, 050098 Bucharest, Romania; 3 Department of Dialysis, University Emergency Hospital Bucharest, Splaiul Independentei nr 168, Sect 5, 050098 Bucharest, Romania; 4 Department of Radiotherapy, Prof. Dr. Al. Trestioreanu Institute of Oncology Bucharest, Șos. Fundeni nr 252, Sect 2, 022328 Bucharest, Romania; 5 National Institute of Neurology and Cerebrovascular Diseases, Șos. Berceni nr 10–12, Sect 4, 041915 Bucharest, Romania; 6 Nephrology and Dialysis Clinic, “Sf. Ioan” Emergency Clinical Hospital, Șos. Vitan-Barzești nr 12, 042122 Bucharest, Romania; 7 Nephrology Clinic, University Emergency Hospital, Splaiul Independentei nr 169, Sect 5, 050098 Bucharest, Romania; 8 Faculty of Dental Medicine, Carol Davila University of Medicine and Pharmacy, Dionisie Lupu nr 37, Sect 2, 020021 Bucharest, Romania

**Keywords:** cardiac valve, atherosclerosis, chronic kidney disease, cytokines, interleukin 6, tumor necrosis factor alpha

## Abstract

*Background and Objectives*: The main cause of morbidity and mortality in hemodialysis patients is cardiovascular disease, which is quite common. The main objective of our study was to investigate the relationship between oxidative stress, inflammation, and vascular and valvular calcifications in hemodialysis patients. *Materials and Methods*: This observational study had 54 hemodialysis patients, with an average age of 60.46 ± 13.18 years. Cardiovascular ultrasound was used to detect and/or measure aortic and mitral valve calcifications, carotid and femoral atheroma plaques, and common carotid intima-media thickness. The aortic calcification score was determined using a lateral abdomen plain radiograph. The inflammatory, oxidative, metabolic, and dietary statuses, as well as demographic characteristics, were identified. *Results*: There were significant correlations between the levels of IL-6 and carotid plaque number (*p* = 0.003), fibrinogen level and aortic valve calcifications (*p* = 0.05), intima-media thickness (*p* = 0.0007), carotid plaque number (*p* = 0.035), femoral plaque number (*p* = 0.00014), and aortic calcifications score (*p* = 0.0079). Aortic annulus calcifications (*p* = 0.03) and intima-media thickness (*p* = 0.038) were adversely linked with TNF-α. Nutrition parameters were negatively correlated with atherosclerosis markers: number of carotid plaques with albumin (*p* = 0.013), body mass index (*p* = 0.039), and triglycerides (*p* = 0.021); number of femoral plaques with phosphorus (0.013), aortic calcifications score with albumin (*p* = 0.051), intima-media thickness with LDL-cholesterol (*p* = 0.042). Age and the quantity of carotid plaques, femoral plaques, and aortic calcifications were linked with each other (*p* = 0.0022, 0.00011, and 0.036, respectively). Aortic annulus calcifications (*p* = 0.011), aortic valve calcifications (*p* = 0.023), and mitral valve calcifications (*p* = 0.018) were all associated with an increased risk of death. *Conclusions*: Imaging measures of atherosclerosis are adversely connected with dietary status and positively correlated with markers of inflammation and risk of mortality.

## 1. Introduction

The majority of hemodialysis patients have cardiovascular disease, which is the most common complication and the leading cause of death in this population [1,2]. In addition to hemodynamic (primarily volume overload) and metabolic complications of chronic kidney disease (malnutrition, mineral and bone disorders, and anemia), cardiovascular disease is also the most common cause of death in this population [1]. The accelerated calcification of cardiac and vascular structures facilitated by the chronic inflammation typical of these patients plays a key role in the pathophysiological pathways linking CKD to cardiovascular disease, which results in a tenfold increase in cardiovascular mortality among hemodialysis patients compared to the general population [2]. Stenvinkel and colleagues have demonstrated that, in patients on hemodialysis, malnutrition, inflammation, and atherosclerotic cardiovascular disease have an increased prevalence and are independently associated with mortality [3]. As a result, the researchers proposed that all three disorders be combined into a single pathophysiological entity known as “MIA syndrome” [4]. It has since been established that the presence of these problems increases the risk of death in dialysis patients, with the detection of MIA syndrome components being critical in the early identification of these high-risk patients [5]. Atherosclerosis, characterized by the accumulation of lipids and immune cells in arterial walls, stands as a leading cause of cardiovascular diseases, including heart attacks and strokes [6]. Emerging evidence highlights inflammation’s critical role in atherogenesis, with TNF-α and IL-6 at the forefront of these investigations [7]. The intricate connection between atherosclerosis markers and pro-inflammatory cytokines, such as tumor necrosis factor-alpha (TNF-α) and interleukin-6 (IL-6), is a complex and highly significant area in cardiovascular research [6]. This issue is crucial because it profoundly impacts our understanding of atherosclerosis development, progression, and potential treatments [7].

This study aims to explore the relationship between vascular and valvular calcifications and the levels of inflammation and oxidative stress in individuals undergoing hemodialysis.

## 2. Materials and Methods

The present study is an observational study performed on 54 patients (32 males, 22 females, age 60.46 ± 13.18 years) on hemodialysis treatment (for 2–84 months, with an average ± standard deviation of 40.87 ± 22.04 months, with an ultrafiltration of 1.94 ± 0.94 L) in the Hemodialysis Department of the University Emergency Hospital Bucharest, Romania. Eight patients died during the following 6 months, seven from cardiovascular causes and one from bronchopulmonary neoplasm.

Cardiac and vascular ultrasound were used to identify and quantify aortic and mitral valve calcifications and carotid and femoral atheroma plaques (including common carotid intima-media thickness), respectively, while lateral abdominal plain radiograph (in which the spine was visible from the eleventh thoracic to the second sacral vertebra) was employed for the calculation of the aortic calcification score as defined by Kauppila [8].

The biological/biochemical parameters were determined in all 54 patients; radiological assessment was performed in 53 patients (one died before the scheduled radiological examination), while ultrasonographic examination was performed in only 51 patients (the other three died before their turn came for ultrasonographic examination). Ultrasonographic and radiological examinations were performed by certified, experienced specialists blinded to the results of lab tests. The aortic calcification score was derived as an average of the estimations given by two independent examiners, each blinded to the assessment of the other.

Fibrinogen, C-reactive protein (CRP), tumor necrosis factor alpha (TNF-α), interleukin-6 (IL-6), and leucocyte count were used as inflammation markers. As malnutrition markers, we have used biochemical parameters (serum levels of albumin, cholesterol, and triglycerides [9]), dietary intake (normalized protein catabolic rate), and body mass index. As oxidative stress markers we have used total antioxidant capacity, oxalate oxidase 1, NADPH oxido-reductase, and xanthine oxidase. For fear of missing some important factor, parameters of bone-mineral metabolism (calcium, phosphorus, calcium × phosphorus product, parathormone) and of liver-biliary function (aspartate aminotransferase, alanine aminotransferase, gamma glutamyl transferase, alkaline phosphatase), anemia-related (hemoglobin, ferritin), coagulation-related (platelet count), and hormonal [dehydroepiandrosterone sulfate (DHEAS)] parameters, electrolytes (sodium, potassium), bicarbonate, glycemia, uric acid, and age were also considered.

The study was designed in compliance with the ethical guidelines of the 1975 Declaration of Helsinki. All participants in this study gave their written informed consent. The study was approved by the local ethical committee (Ethical Committee of Emergency University Hospital Bucharest, 6093/31 January 2020).

At first, univariate analysis (UVA) was performed: the correlations between two categorical parameters (such as gender and mitral valve calcifications) were analyzed by means of Fisher’s exact test; the correlations between one categorical parameter and one numerical parameter were analyzed by Student’s *t*-test (if both sets of numerical values followed a gaussian distribution as assessed by means of Shapiro–Wilk test) or by Mann–Whitney test (if at least one of the two compared sets of numerical values failed to follow a gaussian distribution); the correlations between two numerical parameters were analyzed by computing the correlation coefficient and the associated *t*-statistic (simple regression analysis).

Multivariate analysis (MVA) was then performed, including all the significantly associated parameters (as revealed by UVA) as independent variables.

All the statistical calculations were performed by means of the R language and environment for statistical computing and graphics. The results were considered statistically significant if the *p*-value was below the generally accepted threshold of 0.05.

## 3. Results

Table 1 is a summary of the demographic, biochemical, and imagistic features of our patients.

The vascular access was by tunneled cuffed catheters in 18 patients and by arteriovenous fistula in 36 patients. The primary kidney condition (responsible for the end-stage chronic kidney disease leading to hemodialysis) was: adult type (dominant) polycystic kidney disease (in 5 patients), diabetic nephropathy secondary to type I diabetes mellitus (2), diabetic nephropathy secondary to type II diabetes mellitus (9), focal segmental glomerulosclerosis with nephrotic syndrome (1), IgA nephropathy (1), presumed (not histologically proven) glomerulonephritis (7), congenital renal hypoplasia (1), traumatic or surgical kidney loss (2), drug-induced interstitial nephropathy (1), pyelonephritis due to acquired obstructive uropathy (2), kidney tumor (1), renal vascular disease due to hypertension (21), renal vascular disease due to polyarteritis nodosa (1).

### 3.1. Associations between Imaging Atherosclerosis Markers and Inflammation-Related Parameters

First, the Shapiro–Wilk test was applied to establish whether the statistically analyzed sets of numerical values followed a gaussian distribution (see Table 2).

Positive statistically significant correlations were found between the presence of aortic valve/annulus calcifications and the level of fibrinogen (see Table 3 and Figure S1), between the presence of carotid plaques and the level of IL-6, and between the presence of aortic annulus calcifications and the level of TNF-α (see Table 4 and Figure S2). However, a surprising negative statistically significant correlation was also found between the presence of mitral valve calcifications and cortisol level (see Table 4 and Figure S2). The correlation between the presence of aortic valve/annulus calcifications and the level of CRP was close to the accepted level of statistical significance (see Table 3 and Figure S1). Regression analysis revealed several other positive associations between various imaging atherosclerosis markers (intima-media thickness, number of carotid plaques, number of femoral plaques, total radiologic score of aortic calcifications) and fibrinogen blood level (see Table 5 and Figure S3). Nonetheless, unexpected negative correlations were found between TNF-α level and aortic annulus calcifications (see Table 4 and Figure S2) and intima-media thickness (see Table 5 and Figure S3).

### 3.2. Associations between Imaging Atherosclerosis Markers and Metabolic/Nutrition Parameters

Several statistically significant negative associations were discovered between metabolic/nutritional parameters and the majority of imaging atherosclerotic indicators (see Table 6 and Figure S4).

### 3.3. Associations between Imaging Atherosclerosis Markers and Demographic Parameters

No correlations were found for gender. Nevertheless, age correlated positively with the number of carotid and femoral plaques and the total radiologic score of aortic calcifications (see Table 7 and Figure S5).

### 3.4. Associations between Imaging Atherosclerosis Markers and Other Parameters

No associations were found with the markers of oxidative stress, with the parameters of bone-mineral metabolism and liver-biliary function, with anemia and coagulation-related parameters, with DHEAS, electrolytes, bicarbonate, glycemia, and uric acid. The corresponding results are provided as Supplementary Material.

### 3.5. Associations between Imaging Atherosclerosis Markers and Cardiovascular Death

The Fisher’s exact test indicated connections between cardiovascular death and imaging indicators of atherosclerosis (see Table 8). Simply stated, all deceased patients had calcifications of the aortic valve, annulus, and mitral valve, but only roughly 35–45% of the surviving patients had such calcifications.

### 3.6. Multivariate Analysis

The results of MVA are furnished in Table 9. The complete computations are provided as Supplementary Material.

## 4. Discussion

There is evidence that the amount of atherosclerotic plaques and circulating TNF-α and IL-6 levels are positively correlated, according to numerous studies [10,11]. These results provide credence to the notion that inflammation plays a substantial role in atherosclerosis. Particularly, TNF-α seems to be connected to endothelial dysfunction and the overexpression of adhesion molecules, making it easier for immune cells to be attracted to the vascular wall, which is a critical stage in plaque development [6]. In contrast, research has produced contradictory findings, with some studies suggesting a lack of association or even a negative relationship between the atherosclerotic markers TNF-α, IL-6, and [12,13]. Diverse factors, such as variations in study design, unique patient demographics, and varying measuring procedures, could all contribute to discrepancies between these studies [12]. Additionally, atherosclerosis’s temporal element of inflammation adds intricacy [14]. While acute inflammation may exacerbate the formation of plaque, chronic low-grade inflammation may result from atherosclerosis rather than cause it [14]. Atherosclerosis exhibits inherent diversity, manifesting in various phenotypic forms [15]. This diversity could explain some disparities in study outcomes. Additionally, genetic and epigenetic factors modulating the interplay between inflammation and atherosclerosis remain active areas of research [16,17].

In atherosclerosis, the timing and duration of inflammation must be carefully examined. The inflammatory response is dynamic, comprising distinct phases, each potentially affecting plaque stability and progression differently [14]. Therefore, a thorough analysis should consider the specific stage of atherosclerosis under investigation and the markers used to assess disease severity [14].

The therapeutic implications of these findings are significant. If inflammation indeed drives atherosclerosis, targeting TNF-α and IL-6 could be promising strategies to reduce cardiovascular disease risk [6]. Conversely, if chronic inflammation predominantly results from atherosclerosis, interventions focused on lipid management and plaque stabilization may be more relevant [14].

Patients with CKD, especially those in the later stages, make up a subpopulation in which inflammation is quite important. Chronic inflammation causes the vascular endothelium to undergo smooth muscle cell transition and early atherosclerosis development. Through acute-phase proteins such as CRP or cytokines such as TNF-α and IL-6, chronic inflammation greatly contributes to atherosclerosis in both the tunica intima and tunica media layers of the arterial wall [18,19]. According to a recent study [20], TNF-α and IL-6 are key players in vascular calcification because they have a direct impact on smooth muscle cell remodeling at the vascular level and are linked to both intimal and medial calcification. In individuals who are persistently hemodialyzed, IL-6 affects TNF-α’s vascular effects and is linked to higher rates of cardiovascular and all-cause mortality [20]. Our results showed a positive correlation between imagistic markers of atherosclerosis (intima-media thickness, number of carotid plaques, number of femoral plaques, and aortic calcification score) and plasma fibrinogen level, which is consistent with other researchers’ findings of a link between higher fibrinogen levels and carotid intima-media thickness and valvular and vascular calcifications [21,22]. Moreover, it has been displayed that fibrinogen level is higher in patients with carotid atherosclerosis and is positively correlated with intima-media thickness in patients both on hemodialysis and on peritoneal dialysis, with no significant difference between those on hemodialysis and those on peritoneal dialysis [23,24,25]. Our results demonstrated a statistically significant correlation between aortic valve (both cusps and annulus) calcifications and fibrinogen level, in agreement with the association between high levels of fibrinogen and valvular calcifications demonstrated by other studies [26,27].

On MVA, the association persisted only for intima-media thickness, number of femoral plaques, and aortic calcifications score. For the number of carotid plaques, the association with fibrinogen was effaced by the positive association with age and the negative association with BMI.

Regarding the relationship between CRP and vascular indicators of atherosclerosis in patients with advanced kidney disease, there is no consensus among authors in the literature: Some identified a relationship, while others did not [3,28,29]. Findings from other studies appear to support a synergistic relationship between inflammation and vascular atherosclerosis, which are respectively characterized by higher CRP levels and thicker carotid intima media, in raising cardiovascular and all-cause mortality [30]. In our study, CRP was associated with aortic valve and annulus calcifications, but the corresponding *p*-value was slightly above the generally accepted level of statistical significance. On MVA, age and C-reactive protein were the factors associated with aortic valve and annulus calcifications. This result matches those already published, suggesting that CRP levels positively correlate with coronary artery calcifications in patients with both early and advanced stages of CKD and that in patients on hemodialysis, CRP is associated with valvular calcifications, with aortic valve calcifications leading to aortic stenosis, with arterial calcification score, and in general with cardiovascular (both valvular and vascular) calcifications [22,27,31,32,33]. Moreover, CRP has been shown to be an independent predictor of abdominal aortic calcification in patients on both peritoneal dialysis and hemodialysis [34,35,36].

A higher level of IL-6 was associated with the presence of carotid plaques (at both UVA and MVA) in our patients, which is consistent with the literature, which shows that IL-6 is strongly associated with the severity of carotid atherosclerosis in hemodialysis patients and that increased circulating levels of IL-6 are independently associated with carotid atherosclerosis progression during the first 12 months of dialysis [37,38]. Another study, however, showed that in patients on hemodialysis, the IL-6 level is positively correlated with carotid intima-media thickness but not with the presence of atheroma plaques [37]. The role of IL-6 in the atherosclerotic process was demonstrated in apoE-deficient mice, where recombinant IL-6 injections worsened early atherosclerosis [39]. IL-6 seems to promote atherosclerosis by various metabolic, endocrine, and cellular mechanisms, including increased hepatic synthesis of acute phase reactants, monocyte activation and increased macrophage lipid uptake, lower HDL-cholesterol levels, decreased lipoprotein lipase activity, and activation of the hypothalamic–pituitary–adrenal axis (a potential inducer of obesity, hypertension, and insulin resistance) [40]. IL-6 is involved in the fibrous plaque stage of the atherosclerotic process (Elhage et al., 2001), and high levels of IL-6 are associated with valvular calcifications [26].

Unexpectedly, our findings showed a negative correlation between TNF-α and the occurrence of aortic valve calcifications (cusps and annulus) and carotid intima media thickness. However, the relationships with metabolic variables (uric acid and cholesterol) for aortic annulus calcifications and with fibrinogen for carotid intima media thickness on MVA mask these associations. Some studies have shown a correlation between IL-6 levels and vascular (coronary) calcifications in patients with CKD but not with TNF-α levels; however, other studies have found a significant, albeit weak, correlation between TNF-α levels and the presence of carotid plaques in patients with end-stage renal disease [3,41]. There is a pathophysiological explanation for this association. In vitro studies point out the ability of TNF-α to induce the osteoblastic differentiation of vascular cells and matrix mineralization by calcium incorporation, a process mediated by the protein kinase A-cAMP pathway and resulting in vascular calcification [42].

All 54 patients had their biological and biochemical parameters measured; radiological assessment was performed in 53 patients (one passed away before the scheduled radiological examination), but only 51 patients had their ultrasonograms performed (the other three passed away before their upcoming examination). The association (of both UVA and MVA) between the imagistic markers of atherosclerosis and malnutrition (as reflected by deficient albumin, cholesterol, and triglyceride serum levels, and a lower body mass index) in hemodialysis patients indicated by our results was also demonstrated by other studies [43].

Our findings show a negative relationship between cortisol levels and mitral valve calcification, which contradicts previous findings that cortisol reactivity to stress is associated with the progression of coronary artery calcification and that coronary calcification is associated with a slower decline of cortisol levels throughout the day [44,45,46]. To our knowledge, there is no information in the literature regarding a putative relationship between cortisol and valvular (either mitral or aortic) calcification.

Age was typically the most highly correlated independent factor on MVA, followed by inflammatory markers (fibrinogen or C-reactive protein) and metabolic (malnutrition-related) parameters (cholesterol or BMI), as was expected for the majority of atherosclerosis markers.

The imagistic markers of atherosclerosis were connected to cardiovascular death in our patients. Aortic valve calcifications (both cusps and annulus) and mitral valve calcifications were seen in all patients who passed away within 6 months, although only 35–45% of the survivors exhibited valvular calcifications. These findings are consistent with recent research showing that patients receiving hemodialysis have a higher chance of dying when their aortic calcification score is above 8 [47,48]. The limitations of our study include the small number of patients enrolled and the time lag between the measurement of biochemical parameters and the imagistic investigation (because performing the ultrasound examination of heart valves and carotid and femoral arteries is laborious and time-consuming).

## 5. Conclusions

Atherosclerosis imaging markers are positively connected with inflammation markers and risk of death, but adversely correlated with dietary status. More research (ideally larger, multicentric, longer term, and prospective) is required to better clarify the relationship between atherosclerotic lesions and inflammation in CKD patients, particularly those on hemodialysis.

## Figures and Tables

**Table 1:** Anthropometric, biochemical, radiological, and ultrasonographic features of the patients, # patients = number of patients.

Continuous Parameters (Real Numbers)	# Patients	Mean ± Standard Deviation
Body mass index [kg/m2]	54	27.0 ± 5.95
Albumin [g/dL]	54	3.8 ± 0.52
Cholesterol [mg/dL]	54	154.7 ± 41.05
LDL-cholesterol [mg/dL]	54	82.8 ± 27.41
Triglycerides [mg/dL]	54	128.9 ± 72.03
Fibrinogen [mg/dL]	54	383.9 ± 92.92
C-reactive protein [mg/dL]	54	11.6 ± 20.79
Interleukin-6 [pg/mL]	54	27.0 ± 3.2
Tumor necrosis factor-α [pg/mL]	54	14.2 ± 4.21
Cortisol [µg/dL]	54	94.1 ± 135.08
Intima-media thickness [mm]	51	0.6 ± 0.18
Continuous parameters (integer numbers)		Median (1st–3rd quartile) (minimum–maximum)
Aortic calcifications total score	51	3 (0–6) (0–19)
Carotid plaques	51	4 (1–7) (0–>10)
Femoral plaques	51	>10 (2–>10) (0–>10)
Categorical parameters (calcifications/plaques)		Present (# patients)/ Absent (# patients)
Carotid plaques	51	42/9
Femoral plaques	51	45/6
Mitral valve calcifications	51	24/27
Aortic annulus calcifications	51	22/29
Aortic valve calcifications	51	25/26
Aortic valve/annulus calcifications	51	31/20
Degenerative aortic stenosis	51	7/44

**Table 2:** Results of the Shapiro–Wilk test. A significant (<0.05) *p*-value (bolded) for at least one of the two sets of numbers indicates that Student’s t-test cannot be used and a non-parametric test (such as Mann–Whitney test) should be used instead. Only the pairs of parameters are shown for which subsequently applied tests (Student’s t and Mann–Whitney) yielded statistically significant results. TNF-α = tumor necrosis factor alpha; IL-6 = interleukin 6.

Categorical Parameter	Numerical Parameter	Calcifications/Plaques Present		Calcifications/Plaques Absent		Mann–Whitney Test Needed
		Shapiro–Wilk Statistic	*p* Yielded by Shapiro–Wilk Test	Shapiro–Wilk Statistic	*p* Yielded by Shapiro–Wilk Test	
Mitral valve calcifications	Cortisol	0.96	0.5	0.96	0.4	No
Aortic valve/annulus calcifications	Fibrinogen	0.89	0.003	0.96	0.5	Yes
Carotid plaques	IL-6	0.96	0.1	0.93	0.5	No
Aortic valve/annulus calcifications	C-reactive protein	0.58	3 × 10^−8^	0.6	3 × 10^−6^	Yes
Aortic annulus calcifications	TNF-α	0.93	0.1	0.97	0.5	No

**Table 3:** Associations between imaging atherosclerosis markers (aortic valve/annulus calcifications—a categorical parameter) and inflammatory parameters (continuous numerical parameter) assessed by Mann–Whitney test. # patients = number of patients; q1 = 1st quartile 1; q3 = 3rd quartile.

Categorical Parameter	Continuous Parameter	Calcifications Present		Calcifications Absent		W Statistic	*p*-Value
		# Patients	Median (q1–q3)	# Patients	Median (q1–q3)		
Aortic valve/annulus calcifications	Fibrinogen [mg/dL]	31	389 (331.15–437.15)	20	341.5 (315.45–374.2)	410	0.05
Aortic valve/annulus calcifications	C-reactive protein [mg/dL]	31	5.4 (0.7–14.9)	20	1.65 (0.1–6.15)	405	0.07

**Table 4:** Associations between imaging atherosclerosis markers (aortic valve/annulus calcifications—a categorical parameter) and inflammatory parameters (continuous numerical parameter) assessed by Student’s *t*-test. # patients = number of patients; stdDev = standard deviation; TNF-α = tumor necrosis factor alpha; IL-6 = interleukin 6.

Categorical Parameter	Continuous Parameter	Calcifications/Plaques Present		Calcifications/Plaques Absent		*t-*Statistic	*p*-Value
		# Patients	Average ± stdDev	# Patients	Average ± stdDev		
Mitral valve calcifications	Cortisol [mcg/dL]	24	12.67 ± 3.29	27	15.68 ± 4.47	−2.75	0.008
Carotid plaques	IL-6 [pg/mL]	42	7.26 ± 1.05	9	6.41 ± 0.6	3.32	0.003
Aortic annulus calcifications	TNF-α [pg/mL]	22	7.88 ± 0.81	29	8.44 ± 1.02	−2.21	0.03

**Table 5:** Associations between imaging atherosclerosis markers (aortic calcifications/mitral calcifications/carotid plaques—the dependent variable) and inflammatory parameters (independent variables) assessed by regression analysis. TNF-α = tumor necrosis factor alpha.

Dependent Variable	Independent Variable	Correlation Coefficient (Confidence Interval)	*t*-Statistic	*p*-Value
Intima-media thickness [mm]	Fibrinogen [mg/dL]	0.46 (0.21–0.65)	3.62	0.0007
Number of carotid plaques	Fibrinogen [mg/dL]	0.3 (0.02–0.53)	2.17	0.035
Number of femoral plaques	Fibrinogen [mg/dL]	0.51 (0.27–0.69)	4.14	0.00014
Aortic calcifications total score	Fibrinogen [mg/dL]	0.36 (0.1–0.58)	2.77	0.0079
Intima-media thickness [mm]	TNF-α [pg/mL]	−0.29 [(−0.52)–(−0.02)]	−2.13	0.038

**Table 6:** Associations between imaging atherosclerosis markers (total radiologic score of aortic calcifications/carotid plaques/femoral plaques—dependent variables) and metabolic/nutrition parameters (independent variable) assessed by regression analysis.

Dependent Variable	Independent Variable	Correlation Coefficient [(Confidence Interval)]	*t*-Statistic	*p*-Value
Number of carotid plaques	Albumin [g/dL]	−0.34 [(−0.57)–(−0.08)]	−2.56	0.013
Aortic calcifications total score	Albumin [g/dL]	−0.27 [(−0.5)–0]	−2.00	0.051
Number of carotid plaques	Body mass index	−0.29 [(−0.52)–(−0.02)]	−2.13	0.039
Intima-media thickness [mm]	LDL-cholesterol [mg/dL]	−0.29 [(−0.52)–(−0.01)]	−2.09	0.042
Number of carotid plaques	Triglycerides [mg/dL]	−0.32 [(−0.55)–(−0.05)]	−2.38	0.021

**Table 7:** Associations between imaging atherosclerosis markers (total radiologic score of aortic calcifications/carotid plaques/femoral plaques—dependent variables) and age (independent variable) assessed by regression analysis.

Dependent Variable	Independent Variable	Correlation Coefficient (Confidence Interval)	*t*-Statistic	*p*-Value
Number of carotid plaques	Age [years]	0.42 (0.16–0.62)	3.24	0.0022
Number of femoral plaques	Age [years]	0.51 (0.28–0.69)	4.20	0.00011
Total score	Age [years]	0.29 (0.02–0.52)	2.15	0.036

**Table 8:** Results of the Fisher’s test for the association between cardiovascular death and imaging atherosclerosis markers (IAM). The columns 2–5 contain the number of patients in the four categories: deceased with positive IAM (D+IAM+), deceased with negative IAM (D+IAM−), surviving with positive IAM (D−IAM+), surviving with negative IAM (D−IAM−).

Imaging Atherosclerosis Marker (IAM)	D+IAM+	D+IAM−	D−IAM+	D−IAM−	*p*-Value	Odds Ratio
Aortic annulus calcifications	5	0	17	29	0.011	Infinite
Aortic valve calcifications	5	0	20	26	0.023	Infinite
Mitral valve calcifications	5	0	19	27	0.018	Infinite

**Table 9:** Multivariate analysis (MVA). In the second column, there are the factors independently associated with the markers of atherosclerosis in the first column. The third column contains the estimate, which is the average change in the log odds of the dependent variable associated with a one-unit increase in each independent variable (for the dependent variables influenced by at least two independent variables, according to MVA) and Spearman’s rank correlation coefficient (for the dependent variables influenced by only one independent variable, according to MVA—namely intima-media thickness and aortic calcifications total score). Spearman’s method was used for performing the simple regression analysis as the variables were not normally distributed; no confidence intervals can be calculated by Spearman’s method (as it correlates ranks and not actual numbers).

Dependent Variable	Independent Variable	Estimate	95% Confidence Interval	*t*-Statistic	*p*-Value
Carotid plaques	Triglycerides	−0.0022	[−0.0034, −0.00090]	−3.354	0.002
	IL-6	0.13	[0.041, 0.22]	2.845	0.006
Aortic annulus calcifications	Cholesterol	−0.0051	[−0.0081, −0.0021]	−3.374	0.001
	Uric acid	0.12	[0.028, 0.22]	2.536	0.01
Aortic valve calcifications	Age	0.018	[0.0088, 0.027]	3.833	0.0004
	Cholesterol	−0.0030	[−0.006, −0.00005]	−1.993	0.052
Aortic valve/annulus calcifications	Age	0.02	[0.011, 0.028]	4.418	6 × 10^−5^
	C-reactive protein	0.0079	[0.0021, 0.014]	2.680	0.01
Intima-media thickness	Fibrinogen	0.3	_	3.62	0.0007
Number of carotid plaques	Age	0.02	[0.011, 0.028]	3.546	0.0009
	BMI	0.0079	[0.0021, 0.014]	−2.518	0.015
Number of femoral plaques	Fibrinogen	0.02	[0.011, 0.028]	3.244	0.002
	Age	0.0079	[0.0021, 0.014]	3.252	0.002
Aortic calcifications total score	Fibrinogen	0.35	_	2.77	0.008

## Data Availability

The data underlying this article exist as Excel spreadsheets but cannot be shared publicly due to risk of violating the privacy of individuals that participated in the study, as the names of the participating individuals are included in the database. The data can be shared with interested researchers, on reasonable request to the corresponding author.

## Supplementary Materials

The following supporting information can be downloaded at: https://www.mdpi.com/article/10.3390/medicina59101801/s1, Figure S1: Associations between imaging atherosclerosis markers (aortic valve/annulus calcifications) and inflammatory parameters assessed by Mann–Whitney test; Figure S2: Associations between imaging atherosclerosis markers and inflammatory parameters assessed (*y*-axis) by Student’s *t*-test; Figure S3: Associations between imaging atherosclerosis markers (*y*-axis) and inflammatory parameters (*x*-axis) assessed by regression analysis; Figure S4: Associations between imaging atherosclerosis markers (*y*-axis) and metabolic/nutrition parameters (*x*-axis) assessed by regression analysis; Figure S5: Associations between imaging atherosclerosis markers (*y*-axis) and age (*x*-axis) assessed by regression analysis.
