# Supplementary figures and images for: Cardiovascular Calcifications Are Correlated with Inflammation in Hemodialysis Patients
Source: Medicina (Kaunas). 2023 Oct 10;59(10):1801. doi: 10.3390/medicina59101801 (PMC10608311; doi:10.3390/medicina59101801)

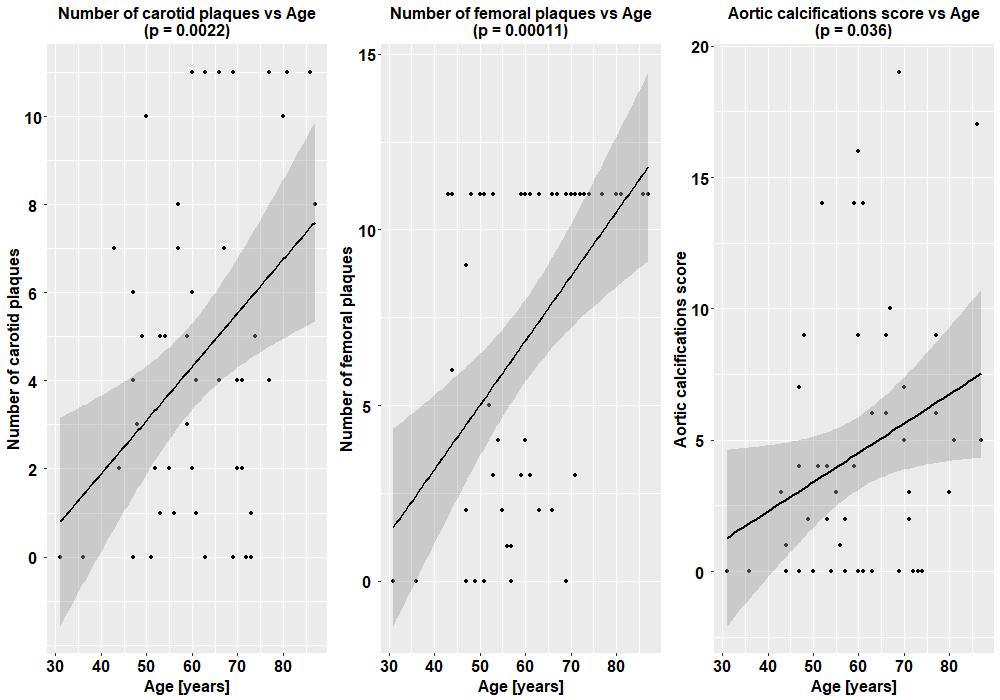

Supplement: Supplementary file 1 [file medicina-59-01801-s001.zip › Figure S5. Associations between imaging atherosclerosis markers (y axis) and age (x axis) assessed by regression analysis..jpg]

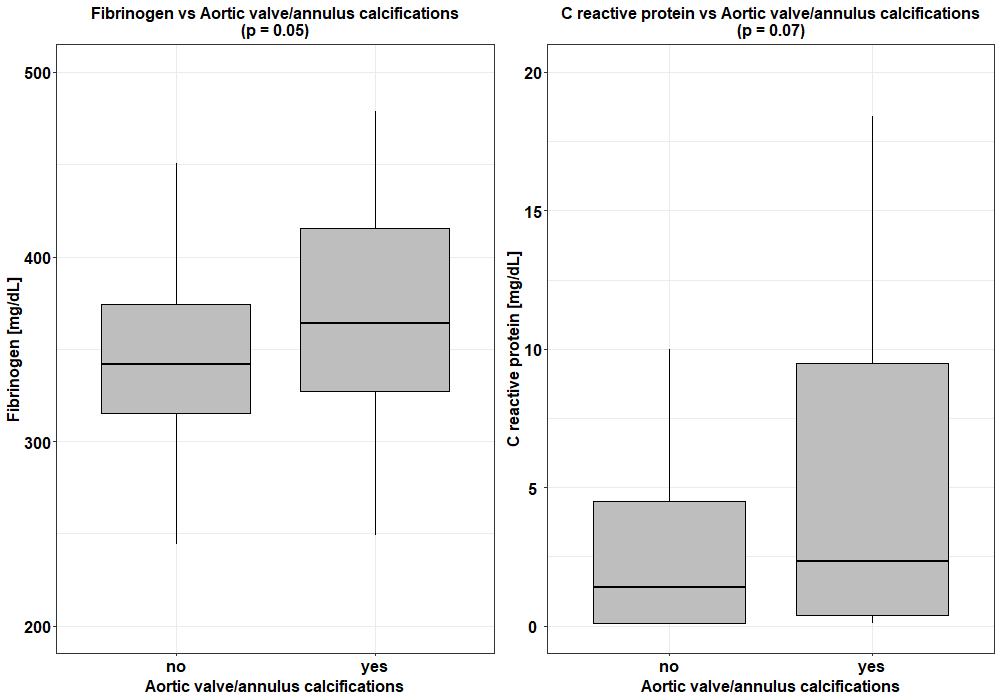

Supplement: Supplementary file 1 [file medicina-59-01801-s001.zip › Figure S1. Associations between imaging atherosclerosis markers (aortic valve annulus calcifications) and inflammatory parameters assessed by Mann-Whitney test..jpg]

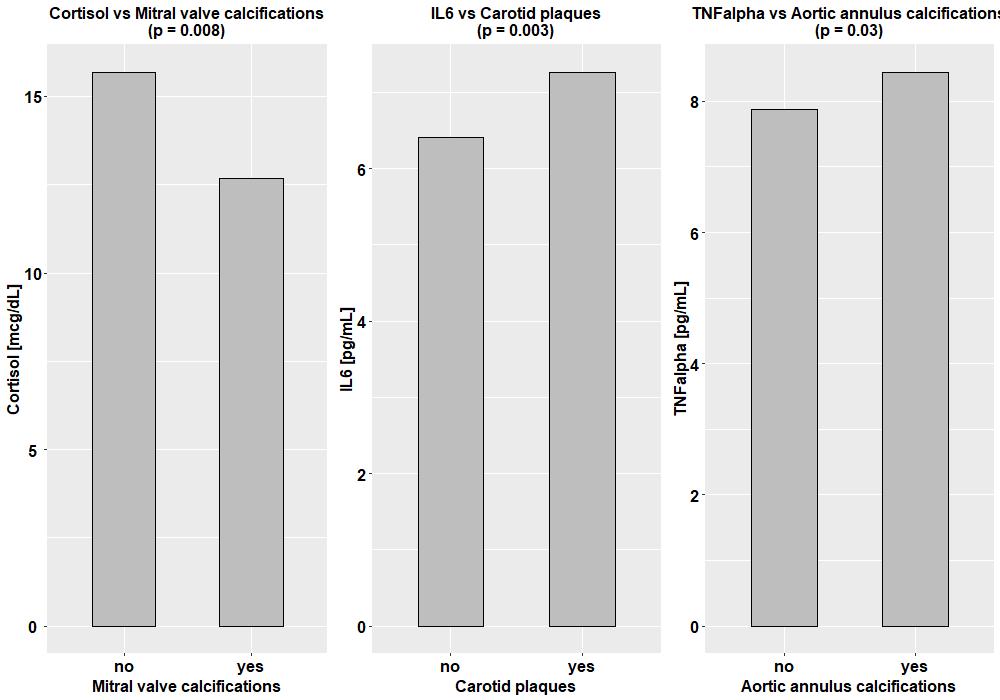

Supplement: Supplementary file 1 [file medicina-59-01801-s001.zip › Figure S2. Associations between imaging atherosclerosis markers and inflammatory parameters assessed (y axis) by Student’s t-test..jpg]

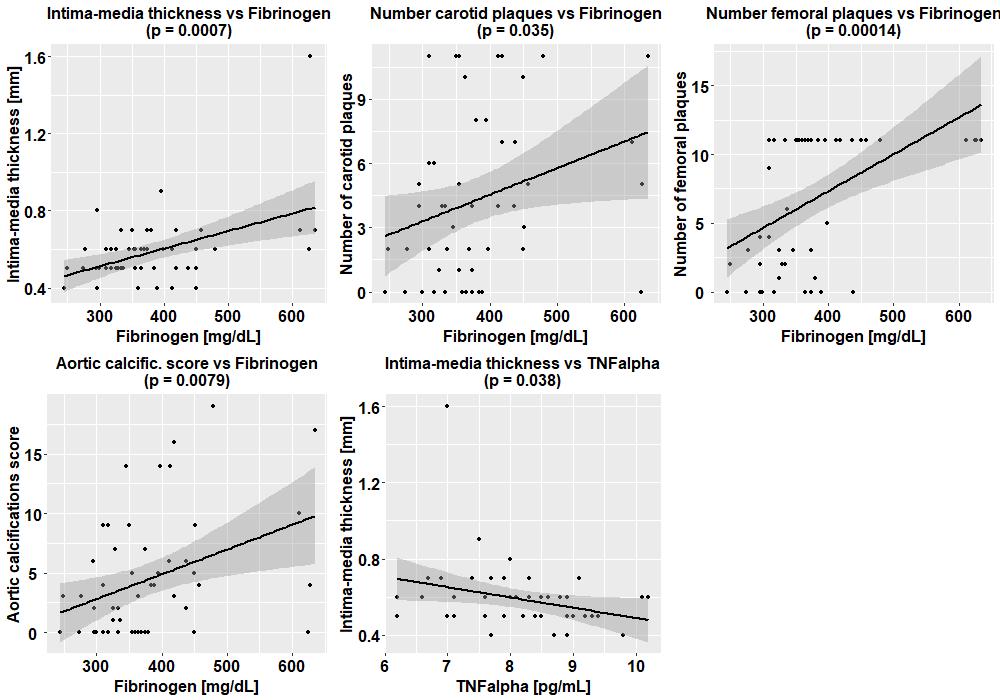

Supplement: Supplementary file 1 [file medicina-59-01801-s001.zip › Figure S3. Associations between imaging atherosclerosis markers (y axis) and inflammatory parameters (x axis) assessed by regression analysis..jpg]

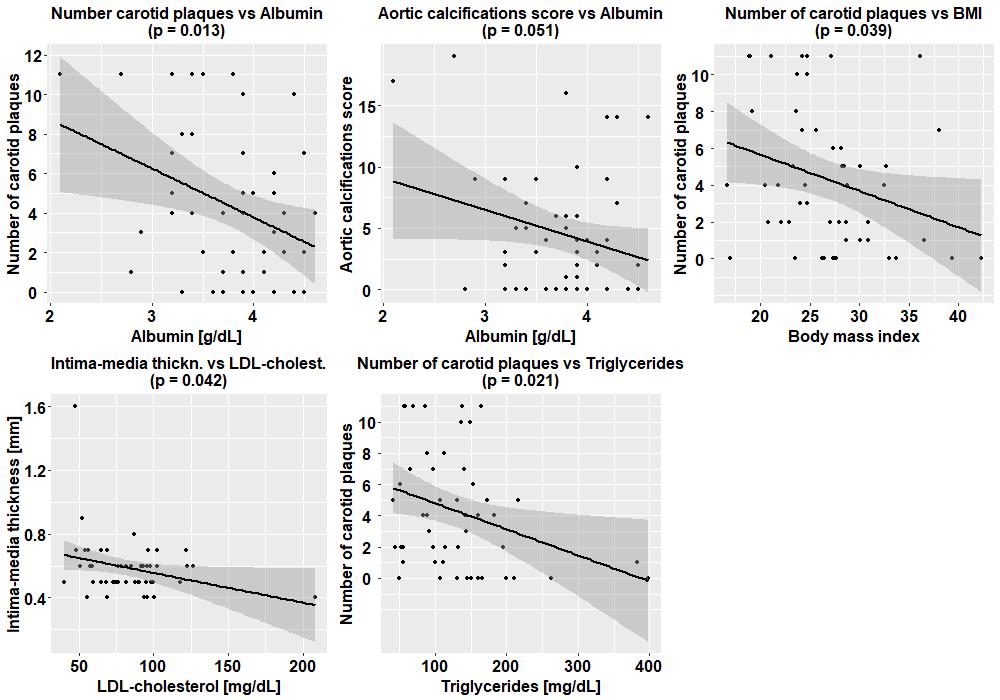

Supplement: Supplementary file 1 [file medicina-59-01801-s001.zip › Figure S4. Associations between imaging atherosclerosis markers (y axis) and metabolic nutrition parameters (x axis) assessed by regression analysis..jpg]
